# Diabetic Cardiomyopathy as a Clinical Entity: Is It a Myth?

**DOI:** 10.7759/cureus.11100

**Published:** 2020-10-22

**Authors:** Mitul P Zaveri, Jamal C Perry, Tayná M Schuetz, Mohammad D Memon, Sadaf Faiz, Ivan Cancarevic

**Affiliations:** 1 Internal Medicine, California Institute of Behavioral Neurosciences & Psychology, Fairfield, USA; 2 Medicine, California Institute of Behavioral Neurosciences & Psychology, Fairfield, USA

**Keywords:** dilated cardiomyopthy, type 2 diabetes mellitus, diabetic cardiomyopathy

## Abstract

Dilated cardiomyopathy (DCM) is a common form of cardiomyopathy that affects the cardiac muscle. It is a life-threatening condition that causes heart failure as it decreases the myocardial ability to pump sufficient blood throughout the body. Numerous causes trigger DCM without pathophysiology; however, the key concept is a decrease in the systolic function of either the left ventricle or of both the left and right ventricles. Long-term diabetes plays an important role in the pathogenesis of DCM in the form of diabetic cardiomyopathy. Diabetic cardiomyopathy is a non-ischemic form of DCM, which is associated with diabetes. It is unrelated to atherosclerosis and hypertension. The PubMed and Google Scholar databases were used to identify the relevant studies related to diabetes and DCM. We found that diabetes was associated with cardiac muscle injury by activating the renin-angiotensin-aldosterone system, myocardial inflammation, and fibrosis. Based on the available data, we concluded that there is strong evidence to support the interrelation of DCM and diabetes.

## Introduction and background

 It is a well-known fact that diabetes is a significant risk factor for heart failure. In 2015, 30.2 million (i.e., 12.2% of) Americans with heart failure had diabetes as a comorbidity [1]. Diabetes gives rise to structural and functional changes in the myocardial tissue by causing metabolic disturbances and cardiac autonomic function impairment. However, it is not known whether diabetes can cause dilated cardiomyopathy (DCM) [2].

DCM is defined as a progressive and irreversible heart muscle defect that causes global contractile (systolic) dysfunction with heart failure. It may be classified as familial (genetic), primary (without any family history), or secondary (associated with diseases such as type 2 diabetes mellitus, Chagas disease, doxorubicin) [3]. In DCM, the cardiac muscle begins to dilate, stretch, and get thinner. This occurs most commonly in adults aged 20-60 years [4]. DCM is one of the most common forms of cardiomyopathy, with a predicted incidence of one in 400 in the United States [3-5].

Based on a statistical analysis conducted from 1975 to 1984, the prevalence of DCM among American and European citizens was one in 2,700 with a male to female ratio of 3:4 [6]. The survival rate of DCM is less than 50% at 10 years [3]. The three-year mortality rate of treated DCM patients remains as high as 12%-20%, with heart failure being the most common cause of death [7].

Diabetic cardiomyopathy is a form of cardiomyopathy associated with diabetes, which causes significant structural and functional changes in the myocardium, not attributed to coronary artery disease and hypertension [8]. It is characterized by myocardial fibrosis, dysfunctional remodeling of the cardiac tissue, and diastolic dysfunction [9].

Since the mortality rate of DCM is relatively high, it becomes essential to identify its secondary causes. We have reviewed the currently available literature for associations between DCM and diabetes using PubMed and Google Scholar databases. The following keywords were used: dilated cardiomyopathy, diabetes mellitus type 2, and diabetic cardiomyopathy.

## Review

Genetics

Alström Syndrome

Alström syndrome is a rare autosomal recessive disorder characterized by childhood obesity, neurosensory deficits, loss of vision, loss of hearing, and diabetes [10-13]. The gene involved in the syndrome interacts with modifier genes and affects a subset of individuals with DCM [11,12]. The Alström syndrome 1 (ALMS 1) gene contains a sequence variation, including four frameshift mutations and two non-sense codons [10]. The genetic mutation responsible for this syndrome was found on short-arm chromosomes 2p(12 to 13) [10,11]. The life span of patients with Alström syndrome rarely exceeds 40 years [12]. There is no specific therapy for this disease, but an early diagnosis can moderate its progression and phenotype forms as well as can increase patients' longevity [12]. Three Turkish sisters were diagnosed with Alström syndrome in 1987 and followed for 20 years [14]. A novel homozygous disease-causing mutation was identified in the codon-c8164 [14]. Genetic analysis showed a premature termination of codon 10 in each of the three affected sisters along with longitudinal progression in the family [14].

**Figure 1:**
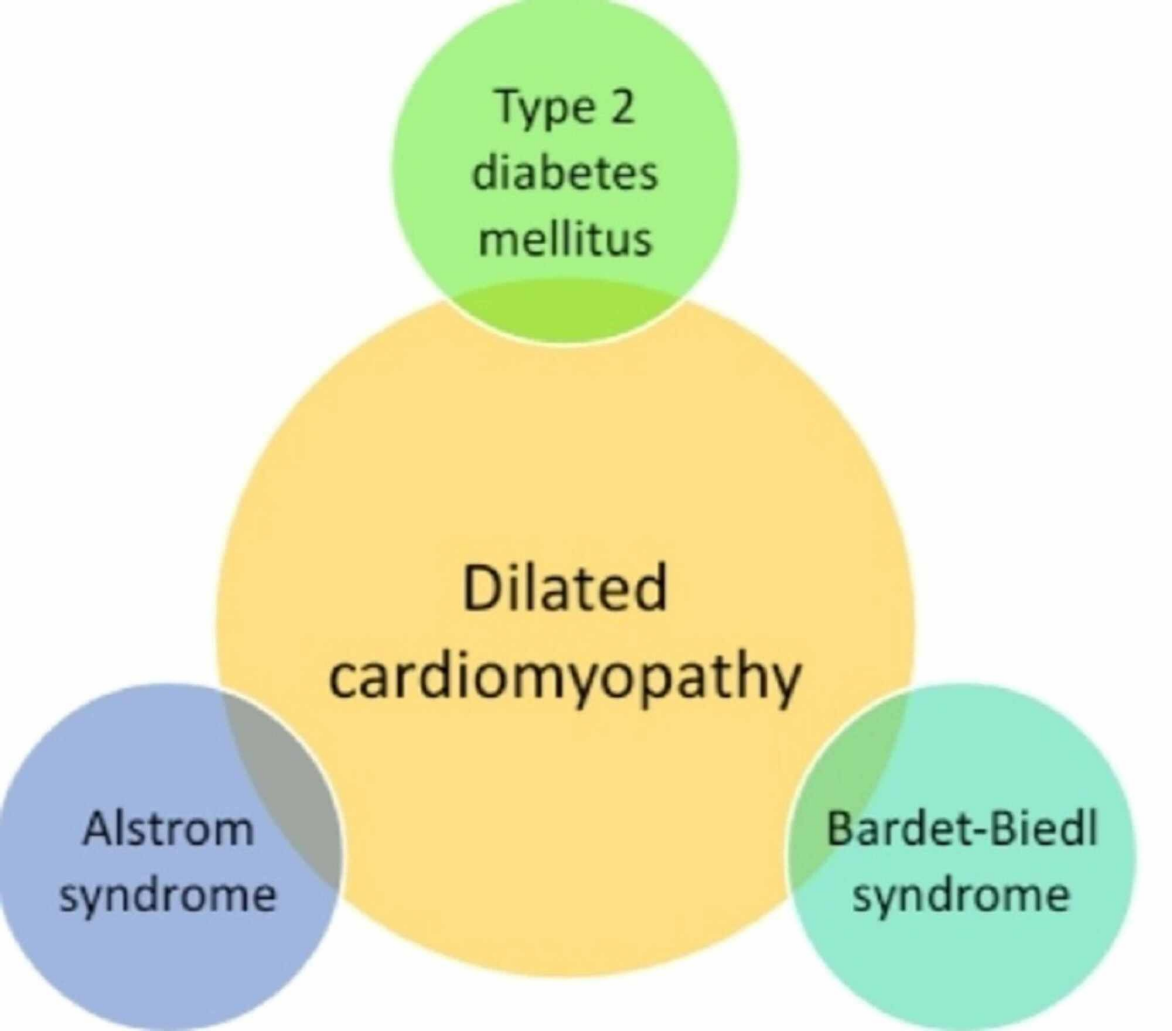
The genetic association of dilated cardiomyopathy and diabetes. This figure summarizes the inter-relationship between dilated cardiomyopathy and diabetes in the form of genetic diseases of Alström and Bardet-Biedl syndromes.

We reviewed five published articles on Alström syndrome in order to identify and correlate the genetic association between DCM and diabetes. As discussed above, a genetic variation in Alström syndrome affects the heart in the form of DCM and also causes metabolic disorders such as diabetes (Figure 1). However, the number of studies on Alström syndrome was insufficient to conclude the relationship between DCM and diabetes.

Bardet-Biedl Syndrome

Bardet-Biedl syndrome is a genetically heterogeneous autosomal recessive disorder characterized by retinitis pigmentosa, polydactyly, central obesity, and secondary amenorrhea [15]. This syndrome is also associated with diabetes mellitus, hepatic fibrosis, and heart diseases such as DCM [15]. Cartinoiu et al. reported a case of a 20-year-old Caucasian male with hearing and vision loss, insulin-resistant diabetes, short stature, and DCM [16]. After examining his medical records, they discovered that two of his relatives had presented with clinical symptoms of Bardet-Biedl syndrome [16]. They concluded that a common unknown genetic link might be responsible for the syndrome as well as DCM [16]. Elbedour et al. performed echocardiographic studies on 22 patients with Bardet-Biedl syndrome [17]. They reported hypertrophy of the interventricular septum in all patients of Bardet-Biedl syndrome with DCM. As a result, DCM was present in 50% of the patients with Bardet-Biedl syndrome [17]. Hence it was concluded that echocardiography should be included along with clinical examination for patients with Bardet-Biedl syndrome [17].

After reviewing three articles on Bardet-Biedl syndrome, we found that DCM and diabetes were phenotypic forms of this syndrome (Figure 1). However, DCM is a rare finding in patients. The number of studies included in the research was limited and was inconclusive in establishing a relation between DCM and diabetes. 

Pathophysiology

Diabetic Cardiomyopathy

**Figure 2:**
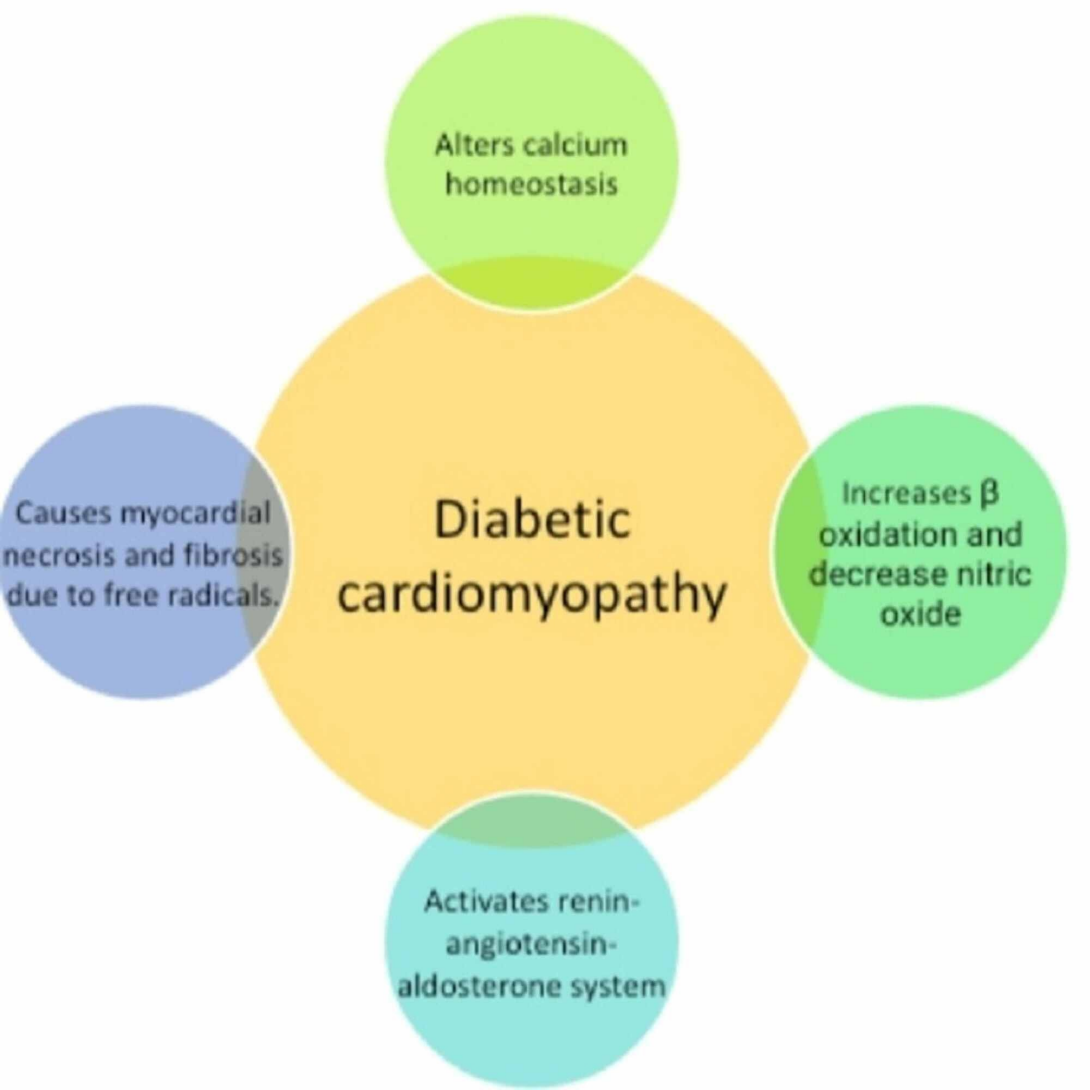
Pathophysiology of diabetic cardiomyopathy. This figure summarizes four different pathophysiologic changes due to diabetic cardiomyopathy in the myocardial tissue by activating RAAS, altering calcium homeostasis, lowering HDL, and increasing free radicals.

Diabetic cardiomyopathy is a distinct disease entity characterized by abnormal myocardial performance or structure in the absence of epicardial coronary artery disease, hypertension, and structural valvular disease [18-21]. Seventy five percent of patients with idiopathic DCM were also found to have diabetes [18]. Microvascular complications of diabetes show the strongest association with DCM [18]. Diabetic cardiomyopathy leads to myocardial fibrosis and myocardial hypertrophy, directly or indirectly affecting renin-angiotensin system activation, cardiac autonomic neuropathy (Figure 2), and alterations in calcium homeostasis [18]. Hyperglycemia due to diabetes mellitus causes myocardial fibrosis and necrosis by increasing the number of myocardial free radicals and oxidants, which in turn decrease nitric oxide (Figure 2) and worsen endothelial function, resulting in myocardial inflammation [19]. Diabetes increases the risk of heart failure four to five times [21]. Diabetic cardiomyopathy represents a distinct structural and functional disorder of myocardium characterized by cardiac hypertrophy and myocardial stiffness [21]. According to Maisch et al., diabetic cardiomyopathy is a poorly understood disease phenomenon [22]. However, several pathologic factors associated with hyperglycemia increase β-oxidation, consecutive free fatty acid damage (lipotoxicity) of the myocardium tissue, renin-angiotensin-aldosterone system activation (RAAS), and altered calcium homeostasis [22]. This leads to myocardial tissue structural changes from the natural collagen network to stiffer matrix and formation of the advanced glycation end product (AGE) [22]. Impairment in glucose transporters, disorders of copper metabolism, and insulin resistance cause myocardial apoptosis and fibrosis [23]. This leads to an increase in angiotensin 2, IGF-1, and pro-inflammatory cytokines and brings about changes in matrix metalloproteinase activity [23]. Spillmann et al. stated that hyperglycemia, hyperinsulinemia, and hyperlipidemia lead to altered cardiac structure and function [24]. It has been demonstrated that low HDL plays a pathogenic role in consequence of diabetes mellitus and indirectly affects diabetic cardiomyopathy [24].

We reviewed nine published articles to understand the role of diabetes in the pathogenesis of DCM. While analyzing the studies, we identified a new disease entity as diabetic cardiomyopathy. Hence, from the above studies, we can deduce that diabetes plays an important role in DCM development in the form of diabetic cardiomyopathy (Figure 2).

Epidemiology

In 2002, a cross-sectional study was conducted on 2042 randomly selected residents of Olmsted County, Minnesota, aged 45 years or above [25]. The objective of the study was to determine the prevalence of diabetic cardiomyopathy and identify its morbidity and mortality rate [25]. All the patients underwent Doppler echocardiography for an assessment of both systolic and diastolic functions [25]. Diabetic cardiomyopathy was diagnosed in patients with systolic or moderate diastolic dysfunction in the absence of coronary artery disease and hypertension [25]. In this study, 23 participants were diagnosed with diabetic cardiomyopathy with a prevalence of 1.1% [25]. Among the diabetic patients, 16.9% met the diabetic cardiomyopathy criteria, and 54.4% had diastolic dysfunction [25]. It was concluded that prevalence was high in the community, and the mortality and morbidity of diabetic cardiomyopathy were relatively high [25]. In 2003, Witteles et al. conducted a clinical trial to quantify the prevalence of abnormal glucose tolerance and insulin resistance in patients with idiopathic DCM [26]. Insulin resistance was considered an independent risk factor for heart failure and ischemic cardiomyopathy [26]. For the study, 230 patients from Stanford University with heart failure were screened for idiopathic DCM in the absence of comorbid medical conditions [26]. An oral glucose tolerance test was conducted on 43 patients who met these conditions, and their plasma glucose was compared with that of 40 healthy volunteers [26]. It was concluded that insulin resistance and abnormal glucose tolerance were more prevalent in patients with idiopathic DCM [26].

The clinical studies showed a strong correlation between DCM and diabetes. However, the number of studies conducted is limited, and diabetic cardiomyopathy is a rarely diagnosed medical condition in clinical practice. Hence, a more detailed clinical analysis is required to establish the relationship between DCM and diabetes.

Limitations

This study has several limitations. First, very little data is available to establish a close link between type 2 diabetes mellitus and DCM. Second, some studies included in this article were conducted over 10 years ago. And finally, in this paper, we were unable to conduct a quality assessment of individual articles due to limited access to some of the articles.

## Conclusions

After reviewing all the available literature to identify the association between DCM and diabetes, we can conclude a strong co-occurrence between the two diseases. Certain studies also highlighted the genetic mutations associated with Alström and Bardet-Biedl syndromes, which resulted in DCM and diabetes. A new disease (diabetic cardiomyopathy) was also identified as a form of idiopathic DCM associated with type 2 diabetes mellitus. Diabetic cardiomyopathy causes unique pathological changes in the myocardium by altering calcium homeostasis, increasing β-oxidation, activating the RAAS, and decreasing nitric oxide. However, diabetic cardiomyopathy needs to be further researched. 
